# *In-vivo* antidiarrheal activity of aqueous and hydroethanolic extracts of *Anacardium occidentale* and *Khaya senegalensis* used in Benin (West Africa) in a castor oil-induced diarrhea model traditionally

**DOI:** 10.3389/fphar.2026.1812204

**Published:** 2026-06-11

**Authors:** Edna Hounsa, Sedami M. Fagla, Eric Agbodjento, Jean Robert Klotoe, Victorien Tamegnon Dougnon

**Affiliations:** 1 Research Unit in Applied Microbiology and Pharmacology of Natural Substances, Research Laboratory in Applied Biology, Polytechnic School of Abomey-Calavi, University of Abomey-Calavi, Abomey-Calavi, Benin; 2 Medicinal Organic Chemistry Laboratory (MOCL), Faculty of Health Sciences, University of Abomey-Calavi, Abomey-Calavi, Benin

**Keywords:** *Anacardium occidentale*, antidiarrheal activity, Benin, *Khaya senegalensis*, protective effect

## Abstract

**Background:**

The management of diarrheal diseases involves the use of medicinal plants, particularly in developing countries, where access to conventional treatments remains limited.

**Methods:**

The study evaluated the *in-vivo* antidiarrheal activity of aqueous and hydroethanolic extracts of *Khaya senegalensis* stem bark and *Anacardium occidentale* leaves using castor oil induced diarrhea model in male Wistar rats.

**Results:**

The hydroethanolic extracts of both plants studied delayed the onset of diarrhea, reduced stool frequency, decreased stool water content, and lowered the purgation index. Particularly, the hydroethanolic extract of *Khaya senegalensis* at 200 mg/kg showed effects similar to Loperamide for certain specific parameters, including the percentage of diarrhea inhibition. However, no reduction in fecal weight was observed. The extracts also showed antioxidant activity associated with their polyphenol and tannin contents.

**Conclusion:**

These findings support the traditional use of *Khaya senegalensis* and *Anacardium occidentale* as an antidiarrheal protective effects may involve modulation of intestinal secretion, motility and oxidative stress. This study provides preliminary experimental evidence supporting the therapeutic potential of these botanical drugs, although further mechanistic investigations are required.

## 1 Introduction

Diarrhea is the second leading cause of death in children under five worldwide and accounts for 1.5 million child deaths per year according to the WHO report updated in 2023 (WHO, 2023). It is characterized by a digestive disorder that consists of the excretion of usually liquid or loose stools at a higher frequency than normal (Behera and Mishra, 2022). Therapy for the management of this disease is based on oral rehydration with electrolyte solution and antibiotic therapy (Ahomadegbe et al., 2021). According to the World Health Organization, antibiotics are particularly sought for the treatment of bloody diarrhea, suspected cholera or associated sepsis (Mekonnen et al., 2018; USAID-UNICEF-WHO, 2005). In recent years, the phenomenon of antimicrobial resistance has contributed to therapeutic failure in the management of infectious diseases (Prestinaci et al., 2015). Regarding diarrhea, the emergence of antibiotic-resistant strains of pathogens has hampered control efforts, particularly in settings where treatment options are limited (Afum et al., 2022). Nowadays, medicinal plants are a valuable source for the discovery of molecules with antimicrobial activities (Katiyar et al., 2012). The World Health Organization (WHO) has recommended the use of medicinal plants for diarrheal diseases via an approach based on traditional medicine practices and prevention approaches (WHO, 2011). Since then, several studies have been conducted on plants with antidiarrheal potential (Njume and Goduka, 2012).

In west African countries, especially, Benin, the flora is very rich in medicinal plants (Akoègninou et al., 2006; WAHO, 2020). *Khaya senegalensis* and *Anacardium occidentale* are two medicinal plants used in the traditional treatment of diarrhea (Dougnon et al., 2021a). In the scientific literature, some studies have documented the antidiarrheal potential of these two plants (Araújo et al., 2015; Elisha et al., 2013; Nwosu et al., 2011). *Khaya senegalensis* is a medicinal plant traditionally used to treat gastrointestinal disorders in various parts of the world (Offiah et al., 2011; Rawat et al., 2017). The decoction of stem bark extract from this plant is indicated in the treatment of mucous diarrhea, malaria, fever, and venereal diseases as well as hookworm (Iwu, 2014).

Various studies have supported the traditional use of *Anacardium occidentale* and *Khaya senegalensis* in the management of diarrhea and other gastrointestinal disorders in Africa and other tropical regions (Offiah et al., 2011; Omoboyowa et al., 2015; Omolaso et al., 2021; Udedi et al., 2013). Different parts of these botanical drugs, including leaves, stem bark, gum, and kernels, are commonly used in traditional medicine for the treatment of acute diarrhea.

In addition to their use in traditional medicine to treat diarrhea*,* these two plants possess a wide range of pharmacological activities and are recognized as rich sources of bioactive plant metabolites. Previous experimental studies and literature reviews have highlighted the antioxidant, anti-inflammatory, antimicrobial, antidiabetic, hepatoprotective, and antiparasitic properties associated with these plant species (Adamu et al., 2022; Salehi et al., 2020; Soares et al., 2022). In particular extracts of *Khaya senegalensis* and *Anacardium occidentale* have demonstrated antioxidant and anti-inflammatory properties in experimental models, notably by reducing oxidative stress and modulating inflammatory mediators and outperforming or approaching standard drugs in some assays (Archana et al., 2021; Heer et al., 2024; Ukpanukpong et al., 2018). To date, no previous study has simultaneously evaluated the *in vivo* antidiarrheal activity, antioxidant potential, and phytochemical composition of aqueous and hydroethanolic extracts of Khaya senegalensis and Anacardium occidentale, which are used in traditional medicine in Benin.

A previous literature review conducted by our research group highlighted the ethnopharmacological relevance and vast therapeutic potential of these medicinal plants in West Africa (Dougnon et al., 2020). However, despite these documented biological properties and traditional uses, experimental data establishing a link between their phytochemical composition, antioxidant activity, and antidiarrheal effects *in vivo* remain limited. In particular, comparative studies evaluating aqueous and hydroethanolic extracts in validated experimental models of diarrhea are rare.

Furthermore, recent work carried out by our research team has provided information on the safety and presence of secondary metabolites such as polyphenols, tannins, flavonoids and leucoanthocyanins in aqueous and hydroethanolic extracts of *K*. *senegalensis* and *A*. *occidentale* L (Dougnon T. V. et al., 2021; Hounsa et al., 2022). Contrary to previous studies, which focused primarily on antibacterial activity, this study provides a comprehensive evaluation of the *in vivo* antidiarrheal effects, antioxidant activity, and phytochemical characteristics of the extracts under investigation. The objective of this study was therefore to evaluate the protective antidiarrheal activity of aqueous and hydroethanolic extracts of *K. senegalensis* and *A. occidentale* using a castor oil-induced diarrhea model, while also assessing their antioxidant activity and phytochemical composition.

## 2 Methodology

### 2.1 Ethics approval statement

This study was approved by the National Health Research Ethics Committee of Benin under number 65/MS/DC/SGM/DRFMT/CNERS/SA and by the Research Unit in Applied Microbiology and Pharmacology of natural substances under number 035-19/URMAPHA/EPAC/UAC. All study methods were performed in accordance with the relevant guidelines and regulations of these ethics committees. The recommendations of these ethics’ committees are in accordance with IACUC (Institutional Animal Care and Use Committee). All experimental procedures and reporting adhered to the ARRIVE (Animal Research: Reporting of *In Vivo* Experiments) guidelines. This ensured transparent and comprehensive documentation of the study design, animal use, methodology, and statistical analysis, thereby enhancing the reproducibility and ethical standards of the research. The animals were examined and adapted to the new environmental conditions for 1 week before the experiment.

### 2.2 Plant material

The plant material consisting of the leaves of *Anacardium occidentale* L (*A. occidentale*) and the bark of the trunk of *Khaya senegalensis* (Desv.) A. Juss. (*K. senegalensis*) was identified at the Herbier National du Bénin under identification numbers YH 434/HNB and YH 435/HNB, respectively. This identification was done by Professor YEDOMONHAN Hounnankpon, Curator of the National Herbarium of Benin. These plants were selected following the ethnopharmacological survey (Dougnon et al., 2021a).

### 2.3 The experimental animals

The experimental animals consisted of three-month-old male Wistar rats weighing between 150 and 180 g, from the animal house of the Institute of Applied Biomedical Sciences (ISBA) of the University of Abomey Calavi.

These rats were acclimatized for 2 weeks in the animal house of the Research Unit in Applied Microbiology and Pharmacology of natural substances according to the recommendation of “Guide for the Care and Use of Laboratory Animals” (Albus, 2012).

### 2.4 Methods

#### 2.4.1 Preparation of the extract

The parts of both plants, collected in their natural habitat, were cleaned with tap water and dried at room temperature at the Research Unit of Applied Microbiology and Pharmacology of Natural Substances. Hydroethanolic and aqueous extracts were prepared from dried powders of *A*. *Occidentale* leaves and *Khaya senegalensis* bark according to the methodology described by. Klotoé et al. (2020). The choice of these types of extracts is based on previous work which demonstrated that these extracts presented the best antibacterial activities on strains involved in the occurrence of diarrhea (Dougnon T. V. et al., 2021). Fifty (50) grams of powder from each plant were separately macerated in 500 mL of solvent, water for the aqueous extract and water-ethanol mixture (V/V) for the hydroethanolic extract. The extracts were concentrated using a rotary evaporator at 40 °C until dry extracts were obtained. The extracts were stored at 4 °C until use. (Klotoé et al., 2020).

#### 2.4.2 Quantitative phytochemical screening

##### 2.4.2.1 Determination of total polyphenol content

Total polyphenol content was determined using the Folin–Ciocalteu method as described by Fanou et al. (2022), with a commercial Folin–Ciocalteu reagent. Quantification was performed based on a calibration curve established with gallic acid (0–200 μg/mL) used as the reference standard. All samples were analyzed in triplicate. Results were expressed as milligrams of gallic acid equivalents per Gram of product.

##### 2.4.2.2 Determination of total flavonoid content

Quantification of total flavonoid metabolites was determined using the aluminum chloride colorimetric method as described by Fanou et al. (2022). This method is based on the formation of a yellow complex between aluminum chloride (AlCl_3_) and the hydroxyl groups of flavonoids, with a maximum absorbance measured at 415 nm. The reaction mixture consisted of the sample solution, 2% AlCl_3_, and ethanol. After incubation for 10 min at room temperature, absorbance was recorded at 415 nm using a spectrophotometer. Quantification was performed using a calibration curve established with rutin (0–1 mg/mL) as the reference standard. All samples were analyzed in triplicate. Results were expressed as milligrams of rutin equivalents per Gram of product.

##### 2.4.2.3 Determination of the content of condensed tannins

Condensed tannin content was determined using the vanillin–hydrochloric acid (vanillin–HCl) colorimetric method (Ali-Rachedi et al., 2018). This assay is based on the reaction between vanillin and flavan-3-ol units under acidic conditions, resulting in the formation of a red-colored complex measurable at 550 nm. The reaction mixture consisted of the sample, 4% vanillin in methanol, and concentrated hydrochloric acid (HCl). Absorbance was measured at 550 nm against a reagent blank. Quantification was performed using a calibration curve prepared with catechin (0–1,000 μg/mL) as the reference standard. All samples were analyzed in triplicate. Results were expressed as milligrams of catechin equivalents per Gram of product.

#### 2.4.3 DPPH radical scavenging antioxidant activity

The method adopted in this study is that applied by Klotoé et al. (2020). The reaction medium consists of different concentrations of the test product and the DPPH ethanol solution. After incubation in the dark for 1 h at room temperature, absorbance readings were taken at 517 nm using a spectrophotometer. The optical densities recorded were used to calculate the percentage of DPPH radical scavenging, which is proportional to the antioxidant capacity of the sample. Ascorbic acid and BHT were used as reference metabolites to assess and compare the antioxidant activity of plant extracts.

#### 2.4.4 Acute toxicity of aqueous and hydroethanolic extracts *Anacardium occidentale* and *Khaya senegalensis*

In our previous study (Dougnon T. V. et al., 2021), we evaluated the acute toxicity of aqueous and hydroethanolic extracts from ten medicinal plants with antidiarrheal properties, including *Anacardium occidentale* and *Khaya senegalensis*. This study followed the OECD guidelines, specifically Guideline 423. A single dose of 2000 mg/kg of body weight was administered to Wistar rats, which were then observed for 2 weeks for 14 days. We monitored various behavioral parameters, such as locomotor activity, respiration, and general condition, while also monitoring for any deaths.

No deaths or signs of toxicity were observed, and we did not detect any significant biochemical, hematological, or histopathological changes. These results suggest that the median lethal dose (LD_50_) of these extracts exceeds 2000 mg/kg.

#### 2.4.5 Design of the animal experiment

**FIGURE 1:**
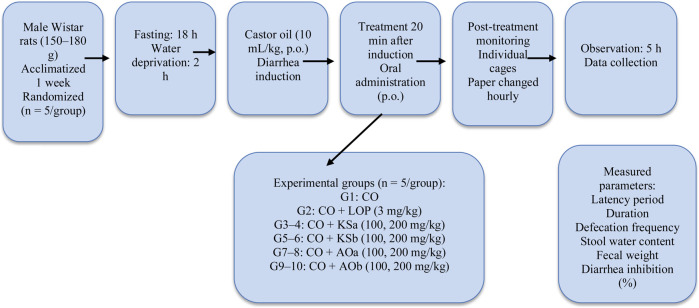
Diarhea’s induction experimental design graphical.

The methodology adopted in this study is that applied by Assiki et al. (Assiki et al., 2022). The induction of diarrhea was performed using castor oil (10 ml/kg) by esophageal gavage. Efficacy tests were performed with aqueous and hydroethanolic extracts of *K*. *senegalensis* and *A*. *occidentale* at doses 100 and 200 mg/kg body weight according to previous studies demonstrating the biological activity of these extracts, including antibacterial effects against diarrheagenic pathogens (Dougnon T. V. et al., 2021; Hounsa et al., 2022). The animals were randomly assigned to the experimental groups. Each group consisted of five rats (positive control group, reference control group, and eight tested group with the extracts). These animals were fasted for 18 h before experimentation and deprived of water 2 h before experimentation. Loperamide used at a dose of 3 mg/kg body weight, served as the reference antidiarrheal drug (Assiki et al., 2022; Schiller et al., 1984). Diarrhea was induced using castor oil (10 ml/kg). After inducing diarrhea with castor oil, treatment should begin 20 min later ». 10 groups of five animals each were fasted for 18 h and treated as follows: group 1, positive control (without treatment) and group 2, control reference LOP (3 mg/kg, b.w., p.o.), and groups 3, 4 KSa (100 and 200 mg/kg, b.w., p. o, respectivly), groups 5,6 KSb (100 and 200 mg/kg, b. w., p.o, respectivly), 7,8 AOa (100 and 200 mg/kg, b.w., p.o, respectivly), and 9,10 AOb (100 and 200 mg/kg, b. w., p. o, respectively (Figure 1). Then, the rats were placed in individual cages under which absorbent paper was spread to collect diarrheal stools. The absorbent paper was changed every hour during the experiment for each rat in the different groups. The rats were observed for 5 h, and different diarrheal parameters were explored.

- The latency period, which measures the time between the administration of castor oil and the appearance of the first diarrheal stools;
- The average mass of diarrheal stools reflects the weight of diarrheal stools obtained during the experiment. The mass of fresh diarrheal faeces collected at the end of each day for each rat in the same lot was added together, and averaged according to the number of rats in each lot (following formula)
- The average mass of diarrheal stools
$$M=\frac{\left(Sum\;of\;fresh\;fecal\;masses\;of\;each\;rat\;in\;a\;lot\right)}{Total\;number\;of\;rats\;having\;diarrhea\;in\;a\;lot}\times 100$$
- Defecation frequency (DF)
$$\text{DF}=\frac{Number\;of\;defecation\;of\;the\;rats\;of\;lot}{Total\;number\;of\;defecation\;of\;all\;the\;lots}\times 100$$
- Percentage of diarrhea inhibition (PDI)
$$\text{PDI}=\frac{\left(Average\;number\;of\;diarrheal\;stools\;of\;control\;lot-Average\;number\;of\;diarrheal\;stools\;of\;test\;lot\right)}{Average\;number\;of\;stools\;of\;control\;lot}\times 100$$
- Water content of diarrhea stools (TES)
$$\text{TES}=\frac{\left(Fresh\;diarrheal\;stool\;mass-dry\;diarrheal\;stool\;mass\right)}{fresh\;diarrheal\;stool\;mass}\times 100$$
- Purge index (PI)
$$\text{PI}=\frac{\left(\%\;of\;rats\;with\;diarrhea\;in\;a\;lot\times average\;number\;of\;diarrheal\;stools\right)}{average\;latency\;period}\times 100$$
  A blind method was not conducted due to logistical constraints, which is acknowledged as a limitation.

#### 2.4.6 Data analysis

The results were presented in the form of graphs, using Graph Pad seven software. Quantitative variables were expressed as mean ± standard deviation. Qualitative variables were expressed as percentages. ANOVA analysis of variance was used to compare the data between different groups. Data were analyzed using SPSS version 26.0 software ANOVA followed by the Tukey *post hoc* test were used. The significance level was set at 5% with P value < 0:05. The Student’s t-test was used to compare the IC_50_ values of each extract with those of each reference standard.

## 3 Results

### 3.1 Quantitative phytochemical screening

Analysis of phytochemical metabolites showed that the levels of polyphenols, flavonoids, and tannins vary depending on the plant species and the solvent used for extraction. Overall, *K*. *senegalensis* exhibited higher polyphenol levels than *A*. *occidentale*, particularly in the hydroethanolic extract (Table 1).

**TABLE 1:** Quantification of polyphenols, flavonoids and tannins in plant extracts.

Plants	Extracts	Polyphenols content (mgEAG/g)	Flavonoides content (mg RuP/g)	Tanins (mg CaP/g)
*Khaya Senegalensis*	Aqueous	26.13 ± 5.35	5.41 ± 0.68	0.68 ± 0.002
	Hydroethanolic	39.43 ± 3.44	4.21 ± 1.8	1.03 ± 0.003
*Anacardium occidentale*	Aqueous	15 ± 0.53	3.21 ± 0.8	0.72 ± 0.003
	Hydroethanolic	17.08 ± 0.29	1.21 ± 0.6	0.92 ± 0.0005

A similar trend was observed for tannins, with the highest value recorded in the hydroethanolic extract of *K*. *senegalensis*. In contrast, the flavonoid content follows a different trend: it is higher in the aqueous extracts for both species, and particularly for *K*. *senegalensis* (5.41 ± 0.68 mg RE/g).

### 3.2 DDPH antiradical activity

DPPH assay showed that antioxidant activity varies considerably depending on the plant species and the extraction solvent used. Overall, hydroethanolic extracts exhibit greater activity, as reflected by lower IC_50_ values (Table 2). Notably, the hydroethanolic extract of *K*. *senegalensis* is the most active (IC_50_ = 0.036 mg/mL), followed by its aqueous extract (IC_50_ = 0.079 mg/mL). Conversely, the extracts of *A*. *occidentale* exhibit more modest antioxidant activity, with higher IC_50_ values, although the hydroethanolic extract remains more active than the aqueous extract.

**TABLE 2:** DPPH radical scavenging activity of plant extracts.

Plants	Extracts	IC50 (mg/mL)
*Khaya Senegalensis*	Aqueous	0.079 ^a^ ^,^ ^b^ ± 0.001
	Hydroethanolic	0.036 ^a^ ^,^ ^b^ ± 0.001
*Anacardium occidentale*	Aqueous	1.53 ^a^ ^,^ ^b^ ± 0.01
	Hydroethanolic	1.18 ^a^ ^,^ ^b^ ± 0.01
Ascorbic acid	—	0.010 ± 0.001
BHT	—	0.63 ± 0.01

a Significantly different from ascorbic acid (P < 0.05).

b Significantly different from BHT (P < 0.05).

Compared to ascorbic acid (IC_50_ = 0.010 ± 0.001 mg/mL), all plant extracts exhibited significantly lower DPPH radical scavenging activity (P < 0.05). Compared to BHT (IC_50_ = 0.63 ± 0.01 mg/mL), *Khaya senegalensis* extracts exhibited significantly higher antioxidant activity, particularly the hydroethanolic extract (IC_50_ = 0.036 ± 0.001 mg/mL) (P < 0.05). In contrast, BHT exhibited significantly better reducing activity compared to the extracts of *Anacardium occidentale* (P < 0.05).

### 3.3 Effect of the different treatments on the latency period

**FIGURE 2:**
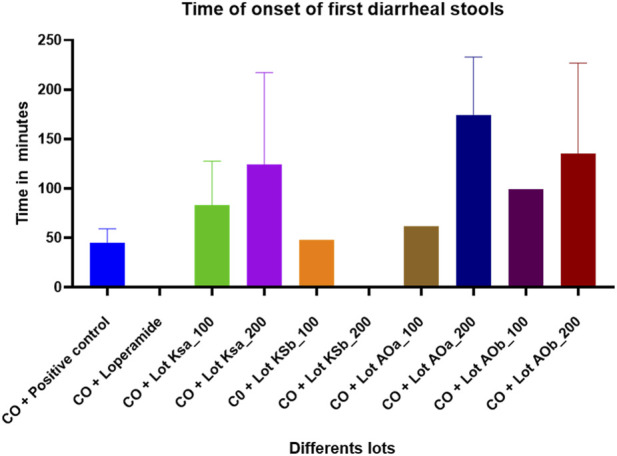
Effect of treatments of aqueous and hydroethanolic extracts of *Anacardium occidentale, Khaya. Senegalensis* and loperamide on castor oil-induced diarrhea on the latency period of diarrhea Legend: AOa, Aqueous extract of *A. occidentale*; AOb, Hydroethanolic extract of *A. occidentale*; KSa, Aqueous extract of *Khaya senegalensis*; KSb, Hydroethanolic extract of *Khaya senegalensis*; 100 = 100 mg/kg dose and 200 = 200 mg/kg dose.

Figure 2 shows the effect of the different treatments on the latency period of the diarrhea. From this figure, it appears that for the positive control, the induced diarrhea occurred 48 min after the administration of castor oil. For reference control lot, no diarrhea stool emission was noted. The same observation was made for the lot treated with hydroethanolic extract of *K*. *senegalensis* at a dose of 200 mg/kg.

Rats from groups treated with aqueous extract of *A*. *occidentale* at 100 mg/kg and hydroethanolic extract *Khaya senegalensis* at 200 mg/kg showed a 2-h delay in the emission of diarrheal stools.

### 3.4 Effect of different treatments on the duration of diarrheal stool output

**FIGURE 3:**
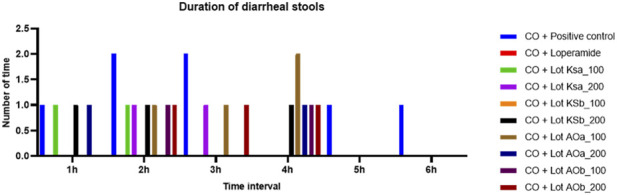
Effect of aqueous and hydroethanolic extracts of *Anacardium occidentale*, *Khaya senegalensis* and loperamide on castor oil-induced diarrhea on duration of diarrheal stools. Legend: AOa, Aqueous extract of *A. occidentale*; AOb, Hydroethanolic extract of *A. occidentale*; KSa, Aqueous extract of *Khaya senegalensis*; KSb, Hydroethanolic extract of *Khaya senegalensis*; 100= 100 mg/kg dose and 200= 200 mg/kg dose.

Figure 3 shows the duration of diarrheal stool emission. The duration of diarrheal stool emission varied between groups. For the positive control lot, diarrheal stool emission continued for up to 6 h of follow-up time. For the experimental groups, the emission of diarrheal stools stopped after 3 h for those treated with *K*. *senegalensis* extracts. On the other hand, for those treated with *A*. *occidentale* extracts, stool excretion stopped after 4 h.

### 3.5 Effect of the different treatments on the defecation frequency of diarrheal feces

**FIGURE 4:**
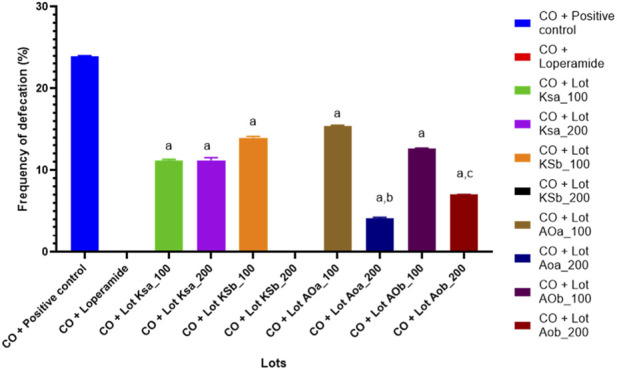
Effect of aqueous and hydroethanolic extracts of *Anacardium occidentale*, *Khaya senegalensis* and loperamide on castor oil-induced diarrhea on defecation frequency of diarrheal feces of rats from different groups Data are expressed as mean ± SD (n = 5). a: significantly different from the positive control; b: significantly different from Lot AOa; c: significantly different from Lot AOb. Legend: AOa, Aqueous extract of *A. occidentale*; AOb, Hydroethanolic extract of *A. occidentale*; KSa, Aqueous extract of *K*. *senegalensis*; KSb, Hydroethanolic extract of *K. senegalensis*; 100 = 100 mg/kg dose and 200 = 200 mg/kg dose.

Figure 4 showed the frequency of defecation of diarrheal feces in rats from different groups. From this figure, we note that the frequency of defecation of diarrheal feces of rats in the positive control lot is significantly elevated in comparison (P < 0.05) to that of rats in the extract-treated groups. Comparative analysis following the two doses tested for each plant extract indicated that rats treated with the aqueous extract of *A*. *occidentale* at 200 mg/kg showed a significant decrease in defecation frequency compared with that obtained at a dose of 100 mg/kg. The same observation was made between the two doses tested for hydroethanolic extract from the same plant.

However, no defecation was recorded for rats treated with loperamide and those treated with hydroethanolic extract of *K*. *senegalensis* at a dose of 200 mg/kg.

### 3.6 Effect of the different treatments on the mass of fresh diarrheal stools

**FIGURE 5:**
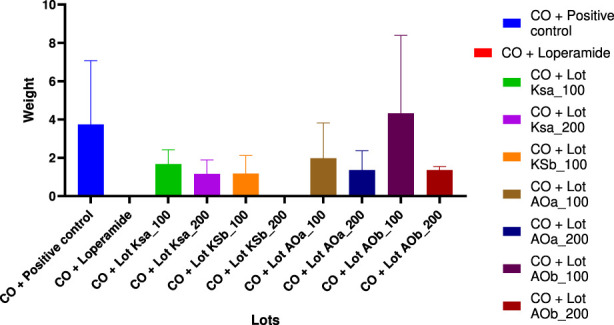
Effect of aqueous and hydroethanolic extracts of *Anacardium occidentale*, *Khaya senegalensis* and loperamide on castor oil-induced diarrhea on the average weight of fresh feces of rats from different groups. Legend: AOa, Aqueous extract of *A. occidentale*; AOb, Hydroethanolic extract of A. *occidentale*; KSa, Aqueous extract of *K. senegalensis*; KSb, Hydroethanolic extract of *K. senegalensis*; 100 = dose of 100 mg/kg and 200 = dose of 200 mg/kg.

Compared with the positive control group, groups treated with extracts of both plants showed no significant decrease in mean fecal weight except for the hydroethanolic extract of *A*. *occidentale* at 100 mg/kg, (P > 0.05) (Figure 5). Like the loperamide-treated group, no diarrheal fresh stool mass was obtained for the groups treated with the hydroethanolic extract of *K*. *senegalensis* at 200 mg/kg b. w. However, despite the absence of changes in fecal weight, other parameters, including, stool frequency, stool water content, and purging index, showed important improvements in the groups treated with the extracts.

### 3.7 Water content of diarrheal stools from the different groups tested

**FIGURE 6:**
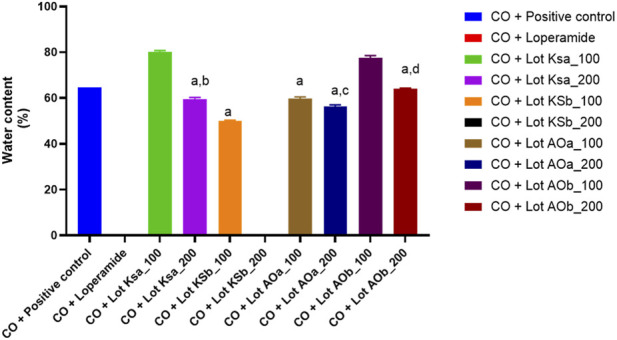
Effect of aqueous and hydroethanolic extracts of *Anacardium occidentale, Khaya senegalensis* and loperamide on castor oil-induced diarrhea on the water content of diarrheic stools of rats from different groups. Data are expressed as mean ± SD (n = 5). a, significantly different from positive control; b, significantly different from KSa lot; c, significantly different from AOa lot; d, significantly different from AOb. Legend: AOa, Aqueous extract of *A. occidentale*; AOb, Hydroethanolic extract of *A. occidentale*; KSa, Aqueous extract of *K. senegalensis*; KSb, Hydroethanolic extract of *K. senegalensis*; 100= 100 mg/kg dose and 200= 200 mg/kg dose.

Figure 6 summarizes the data on the water content of the diarrheal stools. This figure shows that, compared with the control group, a significant reduction (P < 0.05) in the water content of diarrheal stools was achieved for all test groups except those treated with aqueous extract of *K*. *senegalensis* (100 mg/kg) and hydroethanolic extract of *A*. *occidentale* (200 mg/kg). This reduction is dose-dependent except for the two doses of aqueous extract of *A*. *occidentale*.

Like the loperamide group, no diarrhea stool water content could be determined for the lot treated with *K*. *senegalensis* at 200 mg/kg b. w.

### 3.8 Percentage of diarrhea inhibition of the different groups tested

**FIGURE 7:**
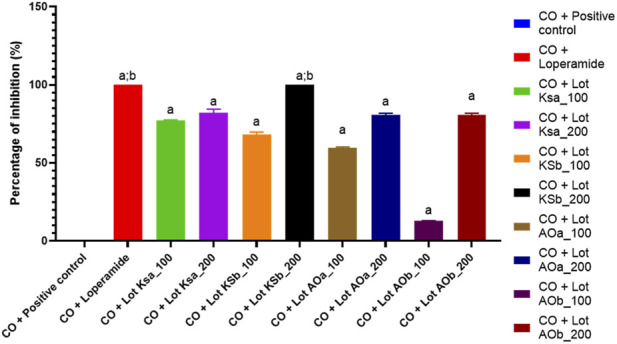
Effect of aqueous and hydroethanolic extracts of *Anacardium occidentale, Khaya senegalensis* and loperamide on castor oil-induced diarrhea on the percentage of diarrhea inhibition of the different groups. Data are expressed as mean ± SD (n = 5). a, significantly different from positive control; b, significantly different from test groups Legend: AOa, Aqueous extract of *A. occidentale*; AOb, Hydroethanolic extract of *A. occidentale*; KSa, Aqueous extract of *K. senegalensis*; KSb, Hydroethanolic extract of *K. senegalensis*; 100 = 100 mg/kg dose and 200 = 200 mg/kg dose.

The data relating to the percentages of inhibition of diarrhea of the various extracts are summarized in As shown in Figure 7, all extracts and loperamide significantly inhibited induced diarrhea compared with the control lot (P < 0.05). However, the diarrhea-inhibiting effect of loperamide was significantly better (P < 0.05) than that of the extracts tested, with the exception of the hydroethanolic extract of *K*. *senegalensis* at a dose of 200 mg/kg bw. Thus, this hydroethanolic extract of *K*. *senegalensis* showed a similar effect to loperamide.

### 3.9 Purge index

**FIGURE 8:**
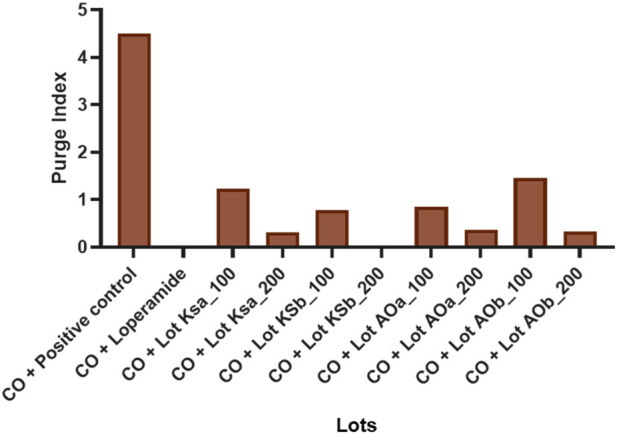
Effect of aqueous and hydroethanolic extracts of *Anacardium occidentale, Khaya senegalensis* and loperamide on castor oil-induced diarrhea on the purge indexLegend: AOa, Aqueous extract of *A. occidentale*; AOb, Hydroethanolic extract of *A. occidentale*; KSa, Aqueous extract of *K. senegalensis*; KSb, Hydroethanolic extract of *K. senegalensis*; 100 = 100 mg/kg dose and 200 = 200 mg/kg dose.

The reduction in the purge index of the extract-treated groups compared to the positive control group is dose dependent (Figure 8). Since there is no diarrheal stool emission for the groups treated with loperamide and hydroethanolic extract of *K*. *senegalensis* at 200 mg/kg b. w., the diarrhea purging index of these groups is zero.

## 4 Discussion

The present study aimed to evaluate the antidiarrheal activity of hydroethanolic and aqueous extracts of *A*. *occidentale* and *K*. *senegalensis* using a castor oil-induced diarrhea model (Assiki et al., 2022; Elisha et al., 2013; Nwosu et al., 2011). The results showed that the tested extracts significantly delayed the onset of diarrhea, reduced stool frequency, decreased stool water content, and lowered the purging index. These results indicate a protective effect of the extracts against experimentally induced diarrhea. These findings constitute preliminary experimental evidence supporting the traditional use of these botanical drugs and highlight their potential value in the management of diarrheal disorders.

Indeed, castor oil contains an active metabolite, ricinoleic acid. This acid, weakly absorbed in the lumen of the small intestine, alters mucosal permeability, altering electrolyte transport (Na^+^ and Cl^−^) and enhancing peristalsis, thereby causing diarrhea (Doe et al., 2019). Consequently, the observed effects of the extracts suggest a possible modulation of these physiological processes. Indeed, the decrease in stool frequency and water content observed in the treated groups may therefore reflect an inhibitory effect on ricinoleic acid-induced intestinal hypersecretion and motility.

The results obtained indicated that the groups treated with *A. occidentale* and *K. senegalensis* extracts showed a consistent improvements in diarrhea-related parameters reduction in the percentage of diarrhea inhibition, water content of diarrheal stools, purging index values. These findings demonstrate the ethnopharmacological uses of the two plants studied in the traditional treatment of diarrhea (Dougnon et al., 2021a). The consistency of these effects on several parameters related to diarrhea further reinforces the reliability of the observed antidiarrheal activity. It should be noted that no significant reduction in fecal weight was observed in most of the treated groups, whereas other parameters showed significant improvements. This suggests that the antidiarrheal activity of the extracts is not uniformly reflected in all measured indicators, underscoring the importance of using multiple complementary parameters when evaluating antidiarrheal effects.

This effectiveness could be explained by the activities of secondary metabolites such as flavonoids, alkaloids, tannins, terpenoids identified in *K. senegalensis* (Abdullahi et al., 2016; Celestine et al., 2017) and *A. occidentale* (Udedi et al., 2013). Phytochemical analysis showed that hydroethanolic extracts, particularly that of *K*. *senegalensis*, contained higher levels of total polyphenols and tannins, which corresponded to their more pronounced antidiarrheal effects observed *in vivo*. Recent studies have shown that polyphenols can modulate intestinal function through antioxidant and anti-inflammatory mechanisms, as well as by reducing intestinal secretion and motility (Ming et al., 2024). Furthermore, experimental studies have shown that polyphenol-rich extracts can significantly delay the onset of diarrhea and reduce its severity in castor oil-induced models (El-Deeb et al., 2025). Tannins, which were more abundant in the hydroethanolic extracts, are known to exert astringent effects that reduce intestinal secretion and strengthen mucosal resistance (Pizzi, 2021; Plaatjie et al., 2024). Although flavonoids were more concentrated in the aqueous extracts, these did not consistently exhibit the highest antidiarrheal activity, suggesting that polyphenols and tannins may play a more significant role in this model. This further confirms the importance of the polarity of the extraction solvent for the extraction of biologically active metabolites.

In addition to their antidiarrheal effects, previous studies conducted have shown that extracts of *A*. *occidentale* and *K*. *senegalensis* exhibit antibacterial activity against the main pathogens responsible for diarrhea and are capable of destabilizing bacterial membranes (Dougnon et al., 2021c). However, this relationship remains correlational, and further studies are needed to elucidate the specific metabolites and mechanisms involved. Therefore, the therapeutic effects observed in this study may involve complementary pharmacological mechanisms.

The best antidiarrheal activity of this study was obtained for the hydroethanolic extract of *K*. *senegalensis* at a dose of 200 mg/kg of body weight. This effect is comparable to that of loperamide effect, the reference antidiarrheal drug used in this study (Parkar et al., 2024; Schiller et al., 1984). Previous studies conducted have shown its effectiveness as a medicinal plant with anti-diarrheal potential (Elisha et al., 2013). The similarity of its effects to those of loperamide further enhances its potential pharmacological relevance. Furthermore, the castor oil-induced diarrhea model suggests that the extracts may exert their antidiarrheal effects through the modulation of intestinal motility, secretion, and inflammatory responses (Doe et al., 2019). These effects appear comparable to those observed with loperamide, the reference antidiarrheal drug used in this study. These findings support the hypothesis that the antidiarrheal effects observed in this study may be linked to both antimicrobial properties and a modulation of intestinal physiology. Tannins may contribute to these effects through their astringent action, which reduces intestinal secretions (Mu’azu and Usman, 2020), while flavonoids and alkaloids may influence intestinal motility and inflammatory responses (Noor and Terefe, 2025; Wang et al., 2021) and contribute to the observed effects. Overall, the results suggest that *K. senegalensis* and *A. occidentale* could be promising candidates for the development of improved traditional medicines for the management of diarrhea.

The results showed that, hydroethanolic extracts exhibited greater DPPH inhibitory activity. These findings suggest that the higher antioxidant activity observed in certain extracts, particularly those rich in polyphenols, may contribute to their antidiarrheal effects, potentially through the modulation of oxidative stress and inflammatory processes in the intestinal mucosa. These findings further support the hypothesis that antioxidant metabolites may contribute to intestinal protection during diarrheal episodes (Wang et al., 2022). In certain models, these factors contribute to the pathophysiology of diarrhea, and the plants studied exhibit both antidiarrheal and anti-inflammatory and antioxidant properties (Afroz et al., 2024; Andargie et al., 2022). The presence of polyphenols, tannins and flavonoids often accounts for both their antioxidant capacity and their protective effects on the intestines (Andargie et al., 2022). While all plant extracts were less active than ascorbic acid, a well-established reference standard, the hydroethanolic extract of *Khaya senegalensis* exhibited significantly higher activity than BHT. Conversely, the extracts of *Anacardium occidentale* proved to be less potent. These differences could be explained by variations in phytochemical composition, particularly by higher levels of polyphenols and tannins observed in the extracts of *Khaya senegalensis* (Cory et al., 2018; Wang et al., 2022). Since oxidative stress contributes to intestinal inflammation and the pathophysiology of diarrhea, the relatively strong antioxidant activity of these extracts may help explain their antidiarrheal effects (Dryden et al., 2006; Jiang et al., 2024).

In conclusion, this study demonstrates that hydroethanolic extracts of *Khaya senegalensis* and *Anacardium occidentale* have protective effects against castor oil-induced diarrhea. These effects are likely due to their antidiarrheal and antioxidant properties, which are associated with their high content of polyphenols and tannins. However, this study remains preliminary, as it did not include in-depth biochemical, enzymatic, or inflammatory analyses. Further studies are therefore needed to elucidate the underlying mechanisms and better evaluate their therapeutic potential in the management of diarrheal diseases.

## 5 Limitations

This study focused on quantifying the main groups of phytochemicals (polyphenols, flavonoids, and tannins) and on assessing antioxidant activity. However.

- Although phytochemical quantification was performed, advanced analytical profiling techniques such as HPLC or LC-MS were not conducted.
- Consequently, the specific bioactive metabolites responsible for the observed antidiarrheal effects were not identified.
- The underlying mechanisms of action therefore remain to be elucidated.

## Data Availability

The original contributions presented in the study are included in the article/supplementary material, further inquiries can be directed to the corresponding author.
